# Histone deacetylase inhibition alters dendritic cells to assume a tolerogenic phenotype and ameliorates arthritis in SKG mice

**DOI:** 10.1186/ar3339

**Published:** 2011-05-18

**Authors:** Kenta Misaki, Akio Morinobu, Jun Saegusa, Shimpei Kasagi, Masaaki Fujita, Yoshiaki Miyamoto, Fumichika Matsuki, Shunichi Kumagai

**Affiliations:** 1 Department of Clinical Pathology and Immunology, Kobe University Graduate School of Medicine. 7-5-2, Kusunoki-cho, Chuo-ku, Kobe 650-0017, Japan; 2 Department of Evidence-based Laboratory Medicine, Kobe University Graduate School of Medicine. 7-5-2, Kusunoki-cho, Chuo-ku, Kobe 650-0017, Japan

## Abstract

**Introduction:**

The purpose of this study was to elucidate the effects of histone deacetylase inhibition on the phenotype and function of dendritic cells and on arthritis in SKG mice.

**Methods:**

Arthritis was induced in SKG mice by zymosan A injection. Trichostatin A, a histone deacetylase inhibitor, was administered and its effects on arthritis were evaluated by joint swelling and histological evaluation. Interleukin-17 production in lymph node cells was determined by an enzyme-linked immunosorbent assay (ELISA). Foxp3 expression in lymph node cells and the phenotypes of splenic dendritic cells were examined by fluorescence-activated cell sorting (FACS). Bone marrow-derived dendritic cells (BM-DC) were generated with granulocyte macrophage colony-stimulating factor. The effects of trichostatin A on cell surface molecules, cytokine production, indoleamine 2,3-dioxygenase (IDO) expression and T cell stimulatory capacity were examined by FACS, ELISA, quantitative real-time polymerase chain reaction and Western blot, and the allo-mixed lymphocyte reaction, respectively.

**Results:**

Trichostatin A, when administered before the onset of arthritis, prevented SKG mice from getting arthritis. Trichostatin A treatment also showed therapeutic effects on arthritis in SKG mice, when it was administered after the onset of arthritis. Trichostatin A treatment reduced Th17 cells and induced regulatory T cells in lymph node, and also decreased co-stimulatory molecule expression on splenic dendritic cells *in vivo*. *In vitro*, trichostatin A markedly suppressed zymosan A-induced interleukin-12 and interleukin-6 production by BM-DC and up-regulated IDO expression at mRNA and protein levels. Trichostatin A-treated BM-DC also showed less T cell stimulatory capacity.

**Conclusions:**

Histone deacetylase inhibition changes dendritic cells to a tolerogenic phenotype and ameliorates arthritis in SKG mice.

## Introduction

Rheumatoid arthritis is a chronic inflammatory disorder, characterized by cellular infiltration of and proliferation in the synovium, leading to the progressive destruction of the joints. Dendritic cells, monocytes, T cells, B cells, and neutrophils infiltrate the synovium and interact with each other to induce chronic synovitis [1,2].

Dendritic cells are efficient antigen-presenting cells, and develop innate and adaptive immune responses through interactions with T cells [3]. Dendritic cells determine the fate of T cell differentiation through the cytokines they produce; IL-12 induces Th1 cells, the combination of IL-6, IL-23, and TGF-β induces Th17 cells, and TGF-β induces regulatory T cells (Treg) [3,4]. Recently, Th17 cells have been shown to play a major role in both human and mouse arthritis [5-7]. Moreover, CD4^+ ^T cells activated by dendritic cells express RANKL and facilitate osteoclast development, leading to bone erosion in joints with rheumatoid arthritis [8]. It is hypothesized that dendritic cells are activated by unknown stimuli in peripheral tissues, and migrate into the lymph nodes, where they induce T cells to proliferate. Activated T cells, as well as dendritic cells, migrate into the joints and induce inflammatory processes, including the production of cytokines such as TNF-α, IL-1, and IL-6, resulting in the characteristically pathological joint damage [9]. In fact, dendritic cells accumulate in the perivascular area in close association with T and B cells in the synovium of joints with rheumatoid arthritis [10]. Thus, dendritic cells are thought to be involved in both initiating and shaping the immune responses in rheumatoid arthritis pathology.

Dendritic cells have been shown to regulate as well as elicit the immune response; those cells with regulatory properties are called tolerogenic dendritic cells. The tolerogenic dendritic cells regulate the immune responses by inducing T cell anergy, inducing Treg, or activating Th2 cells [11]. The characteristics of tolerogenic dendritic cells are as follows: 1) lower expression of cell surface molecules such as CD80 and CD86, 2) a higher expression of indoleamine 2,3-dioxygenase (IDO), 3) decreased secretion of cytokines related to the innate immune response, and 4) lower T cell stimulation capacity [12,13]. Various attempts have been made to generate tolerogenic dendritic cells and endogenous- or *in vitro*-generated tolerogenic dendritic cells have been injected *in vivo *for treating autoimmune disease, illustrating that dendritic cells are now considered as target cells in inflammatory conditions [14].

Histone deacetylase inhibitors (HDAi), such as trichostatin A (TSA) and suberoylanilide hydroximic acid, are small molecule compounds that exert anti-proliferative effects on various tumor cells and are currently used as anti-cancer drug [15]. Histone deacetylase inhibitors are also potential therapeutic agents for rheumatoid arthritis because HDAi suppress joint swelling, synovial inflammation, and subsequent bone and cartilage destruction in animal models of rheumatoid arthritis [16-18]. The mechanism of anti-rheumatic activity by HDAi has been ascribed to the suppression of proliferation and function of synovial fibroblasts. In fact, we have shown the growth-inhibitory effects of HDAi on rheumatoid arthritis-synovial fibroblasts *in vitro *[19]. Recently, however, HDAi have been reported to have immunoregulatory effects along with anti-tumor effects. We and others have shown that HDAi alter the phenotype and cytokine production of dendritic cells, as well as differentiation of monocytes into dendritic cells [20,21].

To clarify the immunoregulatory role of HDAi in a mouse arthritis model, we examined the effect of an HDAi (TSA) on SKG mice, a T cell-mediated model of chronic arthritis. We also examined the effects of TSA on the phenotypes and functions of mouse bone marrow-derived dendritic cells (BM-DC). Here, we show the regulatory effects of TSA on dendritic cells *in vitro*, as well as the preventive and therapeutic effects on arthritis *in vivo*.

## Materials and methods

### Animals

Female SKG mice and female C57BL/6 mice were obtained from CLEA Japan, Inc. (Osaka, Japan). Both the SKG and C57BL/6 mice were housed in the Kobe University animal facility at a constant temperature and were provided laboratory chow and water *ad libitum*. All procedures were carried out in accordance with the recommendations of the Institutional Animal Care Committee of Kobe University.

### Reagents and antibodies

Zymosan A (ZyA), dimethyl sulfoxide (DMSO), phorbol myristate acetate (PMA), suberoylanilide hydroximic acid, ionomycin, bovine serum albumin, 2-mercapto-ethanol (2-ME), and saponin were purchased from Sigma-Aldrich (St. Louis, MO, USA). Phosphate-buffered saline was from Nissui Pharmaceutical Co., Ltd. (Tokyo, Japan). Trichostatin A (TSA), 4% paraformaldehyde phosphate buffer solution, hematoxylin and eosin, RPMI-1640 with L-glutamine, phenol red, and HEPES were from Wako Pure Chemical Industries, Ltd. (Osaka, Japan). EDTA (Dojindo Laboratories, Kumamoto, Japan), fetal bovine serum (MP Biomedicals, Inc., Illkirch, France), 1% penicillin-streptomycin (Lonza Walkersville, Inc., Walkersville, MD, USA), recombinant murine granulocyte macrophage colony-stimulating factor (Peprotech, Rocky Hill, NJ, USA) were also purchased. The PE-anti-mouse FOXP3 Flow kit and allophycocyanin (APC)/Cy7-anti-mouse CD8a were purchased from BioLegend, San Diego, CA, USA. Phycoerythrin (PE)-anti-mouse CD80, PE-anti-mouse CD86, PE-anti-mouse CD40, PE- anti-mouse MHC class II (I-E(k)), fluorescein isothiocyanate (FITC)-anti-mouse B220, FITC-anti-mouse CD25, FITC-anti-mouse CD80, FITC-anti-mouse CD86, FITC-anti-mouse CD40, FITC-anti-mouse MHC class II (I-A/I-E), FITC-anti-mouse CD54, allophycocyanin (APC)-anti-mouse CD11c, and APC-anti-mouse CD4 were purchased from eBioscience (San Diego, CA, USA).

### Induction of arthritis

SKG mice that were seven or eight weeks old were intraperitoneally injected with 2 mg/mouse ZyA, as previously described [22]. Briefly, ZyA suspended in saline was kept in boiling water for 10 minutes and the ZyA solution (0.5 ml/mouse) was intraperitoneally injected into SKG mice. Arthritis developed between 14 and 21 days after injection.

### Treatment of SKG mice with trichostatin A

Trichostatin A (8 mg/kg) was dissolved in DMSO and subcutaneously administered to SKG mice from Day 14 (before the onset of arthritis) to Day 22 (after the onset of arthritis). DMSO was used as a control.

### Evaluation of arthritis

Arthritis severity was evaluated according to the clinical arthritis scores as follows: 0, no joint swelling; 0.1, swelling of one finger joint; 0.5, mild swelling of wrist or ankle; 1.0, severe swelling of wrist or ankle, as previously reported [23]. Arthritis scores for all the digits of the forepaws and hind paws, as well as for the wrists and ankles, were totaled for each mouse. The maximum possible clinical arthritis score is 5.8.

### Histology

Mice were killed on Day 35 after the administration of ZyA. Control mice injected with DMSO were killed at the same time. After the groups of mice were killed, their hind paws were removed, fixed in 4% paraformaldehyde in phosphate-buffered saline, decalcified in EDTA, embedded in paraffin, and sectioned. The samples were then stained with hematoxylin and eosin. Histologic evaluation was performed by the scoring system described previously, in which 0 = no inflammation, 1 = slight thickening of the synovial cell layer and/or some inflammatory cells in the sublining, 2 = thickening of the synovial lining, infiltration of the sublining, and localized cartilage erosions, and 3 = infiltration in the synovial space, pannus formation, cartilage destruction, and bone erosion [24].

### IL-17 production and Foxp3 expression in SKG mice

Inguinal lymph node cells (1.0 × 10^6 ^cells/ml) were collected and stimulated with PMA + ionomycin and the IL-17A levels in the supernatants were determined by an enzyme-linked immunosorbent assay (ELISA) (SABiosciences, Frederick, MD, USA), following the manufacturer's instructions. Inguinal lymph node cells from both the DMSO-treated and TSA-treated SKG mice were collected on Day 35 and then were ground using the inner cylinder of a syringe on the cell strainer (BD Biosciences Pharmingen, San Jose, CA, USA) in a 3.5-cm Petri dish. The cells were stained with APC-anti-CD4, FITC-anti-CD25, and PE-intracellular Foxp3 monoclonal antibodies according to the manufacturer's protocol. Foxp3 expression on gated CD4^+^CD25^+^T cells was determined by flow cytometry.

### Analysis of conventional splenic dendritic cells in SKG mice with fluorescence-activated cell sorting

Splenic cells from both the DMSO-treated and TSA-treated SKG mice were collected on Day 35 and treated with ACK lysing buffer (Lonza Walkersville, Inc.) to lyse red blood cells at 4°C for five minutes, followed by washing twice with 0.5% bovine serum albumin in phosphate-bufferd saline. The cells were incubated with the indicated monoclonal antibodies (FITC-anti-B220, APC-anti-CD11c, APC/Cy7-anti-mouse CD8a and PE- anti-CD80 or PE-anti-CD86 or PE-anti-CD40 or PE-anti-MHC class II) for 30 minutes at 4°C. Isotype-matched antibodies were used as controls, and Fc block (BD Biosciences Pharmingen) was used to block non-specific binding to Fc receptors. After extensive washing, the cells were stained with 7AAD (BD Biosciences Pharmingen). The cells were analyzed on a FACSCalibur or a FACS Canto II flow cytometer (Becton Dickinson, San Jose, CA, USA) at the CD11c ^high^-B220 ^negative ^gate to define conventional dendritic cells. Data were expressed as the mean fluorescence intensity and/or as the percentage (%) of positive cells after subtraction of background isotype-matched values.

### Generation of bone marrow-derived dendritic cells

Bone marrow-derived dendritic cells (BM-DC) were generated from SKG mice. Briefly, bone marrow cells were collected from the SKG mouse femur, and 1.0 × 10^6 ^bone marrow cells were cultured in a 24-well plate with RPMI-1640 supplemented with 10% fetal bovine serum, 1% penicillin-streptomycin, 100 μM 2-ME, and 50 ng/ml recombinant murine granulocyte macrophage colony-stimulating factor. The medium were changed every two days. On Day 8, weakly adherent cells were harvested using 4°C PBS as BM-DC [25].

### Cell surface molecules of bone marrow-derived dendritic cells

Bone marrow-derived dendritic cells were generated as mentioned above and stimulated with ZyA (5 μg/ml), TSA (20 nM), or ZyA+TSA for the last 48 h. Cells were harvested and incubated with the indicated monoclonal antibodies (APC-anti-CD11c and FITC-anti-MHC class II, FITC-anti-CD54, FITC- anti-CD80, FITC-anti-CD86, or FITC-anti-CD40) for 30 minutes at 4°C. Cells were stained and analyzed at the CD11c ^high ^gate using the FACSCalibur as previously described.

### Enzyme-linked immunosorbent assay

Bone marrow-derived dendritic cells (1.0 × 10^6 ^cells/ml) were stimulated with ZyA (5 μg/ml), TSA (20 nM), or ZyA + TSA for 18 h and the IL-12p70, IL-12p40, and IL-6 levels in the culture supernatant were measured with commercial ELISA kits (BD Biosciences, San Diego, CA, USA) following the manufacturer's instructions.

### Quantitative real-time polymerase chain reaction

Levels of IDO1 and IDO2 mRNA expression were determined by quantitative real-time polymerase chain reaction. Total RNAs were isolated using an RNeasy Mini kit (Qiagen, Tokyo, Japan) and cDNA synthesis was performed using Super Script III First-Strand Synthesis System for RT-PCR (Invitrogen, Carlsbad, CA, USA). Amplification was run in triplicate using an SYBR Green Gene Expression Assay (Qiagen) according to the manufacturer's protocol. The primer pairs used in the reactions were purchased from Qiagen (QT00103936 for IDO1 and QT01066345 for IDO2). The amplification reactions, data acquisition, and analyses were performed with the ABI Prism 7900 HT instrument (Applied Biosystems, Foster city, CA, USA). Glyceraldahyde-3-phosphate dehydrogenase (GAPDH, Qiagen QT01658692) was used as the housekeeping gene against which all of the samples were normalized for differences in the amount of total RNA added to each cDNA reaction and for the variation in the reverse transcriptase efficiency among the different cDNA reactions.

### Western blot analysis

Bone marrow-derived dendritic cells were harvested after stimulation with ZyA (5 μg/ml), TSA (20 nM), or ZyA + TSA for 48 h and lysed with RIPA buffer (Thermo Scientific, Rockford, IL, USA) containing protease inhibitor cocktail (Roche Diagnostics, Mannheim, Germany) at 4°C for 30 minutes. After centrifugation at 12,000 × *g *for 15 minutes, the supernatants were removed and the protein concentrations were determined using the BCA Protein Assay Reagent (Pierce Chemical Company, Rockford, IL, USA). Samples containing 10 to 30 μg of proteins were boiled for five minutes in sodium dodecyl sulfate sample buffer (Wako Pure Chemical Industries, Ltd.). The expression of IDO was determined by immunoblot analysis using purified anti-mouse IDO antibody (BioLegend).

### Allo-mixed lymphocyte reaction

Naïve CD4^+^T cells from C57BL/6 mice were purified by positive selection using anti-CD4^+^CD62L magnetic beads (Miltenyi Biotec, Bergisch Gladbach, Germany).

Bone marrow-derived dendritic cells from SKG mice were harvested and purified by positive selection using anti-CD11c+ magnetic beads (Miltenyi Biotec,).

Naïve CD4^+^T cells (1.0 × 10^5^/200 μl) were co-cultured with 2.0 × 10^4 ^control dendritic cells, ZyA (5 μg/ml) dendritic cells, TSA (20 nM) dendritic cells, or ZyA + TSA dendritic cells derived from SKG mouse bone marrow. On Day 5, cell proliferation was determined by a cell proliferation ELISA kit (Roche, Penzberg, Germany), using BrdU and anti-BrdU antibodies.

### Statistical analysis

Results are expressed as the mean ± standard error of the mean (SE). Statistical comparisons were performed by Student's *t*-test. Differences were considered significant when *P *< 0.05.

## Results

### Preventive effects of trichostatin A on SKG mice

**Figure 1:**
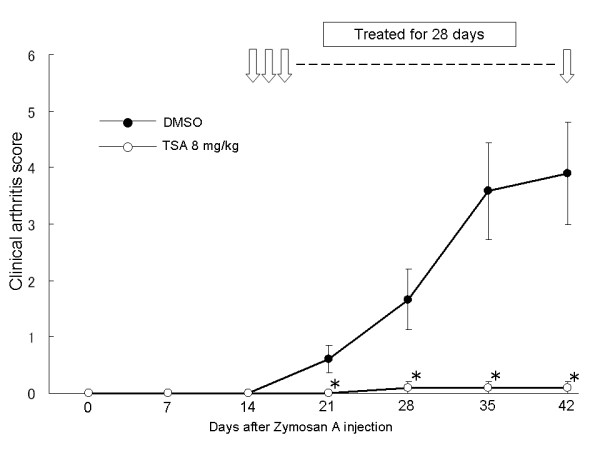
**The effects of trichostatin A on SKG mice**. Zymosan A was administered to SKG mice. Dimethyl sulfoxide or trichostatin A was injected subcutaneously daily for 28 days (Day 14 through Day 42). The clinical arthritis scores were evaluated and the results are expressed as the mean ± SE (DMSO group: *n *= 5, TSA group: *n *= 5). * *P *< 0.05.

We initially examined the preventive effects of TSA on arthritis in SKG mice, an animal model of chronic arthritis that shows a pathology similar to that of rheumatoid arthritis. Zymosan A was administered to SKG mice on Day 0 and DMSO (*n *= 5) or TSA 8 mg/kg (*n *= 5) was subcutaneously injected from Day 14 through Day 42 (for 28 days of treatment). The clinical arthritis scores of the TSA-treated groups were significantly lower than those of the DMSO-treated groups, indicating the preventive effects of TSA on arthritis in SKG mice (Figure 1).

**Figure 2:**
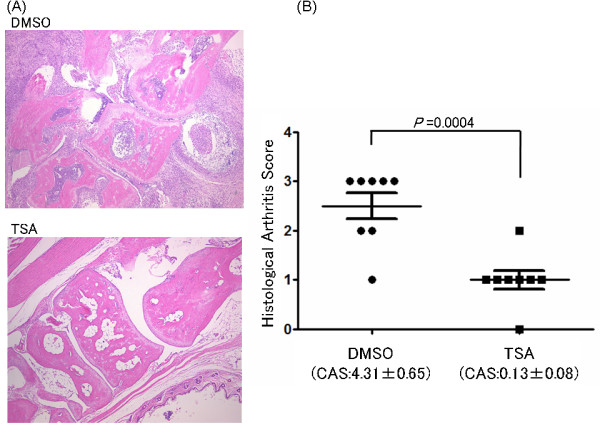
**Histological analysis of SKG mice on Day 35 (after 21 days of treatment)**. (**A**) The histological analysis was performed on their hind paw sections stained by hematoxylin and eosin. Tissues are shown at ×40 magnification. Representative results are shown. (**B**) Histological arthritis scores between the dimethyl sulfoxide- and trichostatin A-treated groups of SKG mice. Mice were killed on Day 35 (treatment for 21 days) and the histological arthritis scores were calculated on their left hind paw. Results are expressed as the mean ± SE (DMSO group: *n *= 8, TSA group: *n *= 8, *P *= 0.0004). CAS, clinical arthritis scores.

We next examined the histological differences between the TSA-treated and control groups. Mice were killed on Day 35 (treatment for 21 days). In the control group, synovial hyperplasia and erosion of articular cartilage and bone were more severe than in the TSA-treated group, as depicted in Figure 2A. The comparison of the histological arthritis scores between these groups clearly showed again the preventive effects of TSA on arthritis in proportion to the clinical arthritis scores (*P *= 0.0004) (Figure 2B).

### The effects of trichostatin A on IL-17 production and Foxp3 expression by inguinal lymph node cells

**Figure 3:**
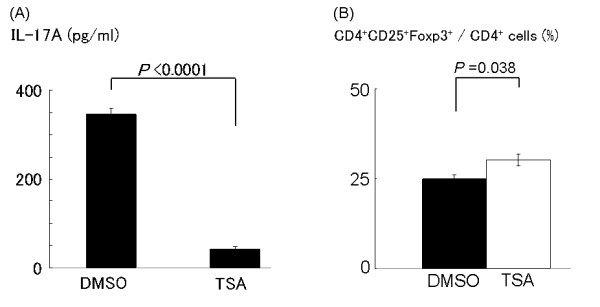
**Production of IL-17A and expression of Foxp3 by inguinal lymph node cells of SKG mice**. (**A**) Inguinal lymph node cells of SKG mice in each group were collected on Day 35. Cells were stimulated with phorbol myristate acetate/ionomycin and the supernatants were collected after 8 h for the measurement of IL-17A. Values are presented as the mean ± SE (DMSO group: *n *= 3, TSA group: *n *= 3, *P *< 0.0001). (**B**) The expression of Foxp3 in inguinal lymph node cells in SKG mice. Inguinal lymph node cells of SKG mice were collected on Day 35 in each group as previously described. Cells were stained for anti-CD4, anti-CD25, and Foxp3. The percentage of CD4^+^CD25^+^Foxp3^+ ^cells among gated CD4^+ ^cells was determined. Results are expressed as the mean ± SE (DMSO group: *n *= 4, TSA group: *n *= 4, *P *= 0.038).

We next examined the effect of TSA on IL-17A production, because IL-17 plays a central role in the induction of severe arthritis in SKG mice [26]. Inguinal lymph node cells from SKG mice were stimulated with PMA/ionomycin and IL-17A in the supernatant was determined by ELISA. IL-17A production by lymph node cells in the TSA group was remarkably reduced compared with the control group (Figure 3A), demonstrating that TSA suppresses the development of Th17 cells *in vivo *in SKG mice.

We also examined whether TSA affected the Treg population in SKG mice. Foxp3 expression in inguinal lymph node cells on Day 35 from the control- and TSA-treated SKG mice were determined by fluorescence activated cell sorting (FACS). We found significant increase in the ratio of CD4 ^+ ^CD25 ^+ ^Foxp3^+ ^cells among CD4^+ ^cells in TSA-treated group compared to control group, suggesting that Treg are involved in the prevention of arthritis in SKG mice with TSA (Figure 3B).

### The effects of trichostatin A on the phenotype of splenic dendritic cells

**Figure 4:**
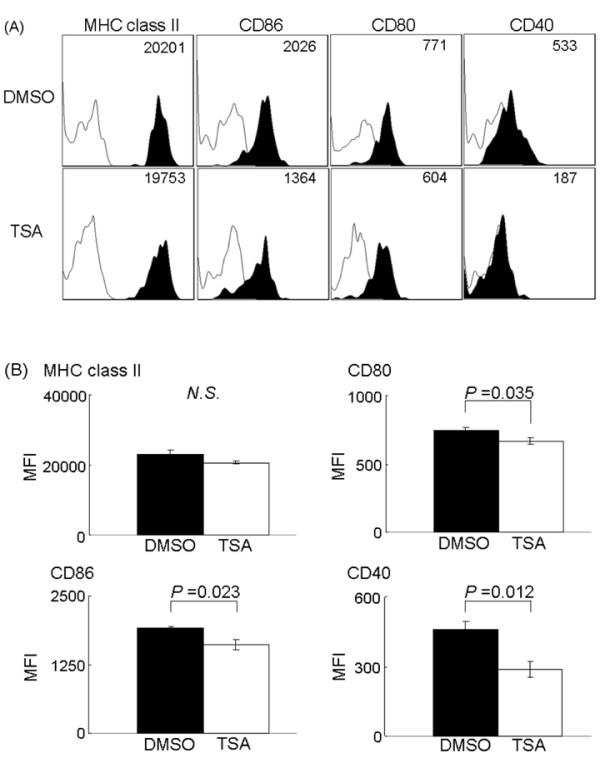
**MHC class II, CD86, CD80, and CD40 expression on CD8α^+ ^splenic conventional dendritic cells**. Spleen cells were isolated from dimethyl sulfoxide- or triclostatin A-treated SKG mice on Day 35 and stained for each marker. Gates were set on CD8α^+ ^conventional dendritic cells and cell surface molecules were analyzed on fluorescence-activated cell sorting. (**A**) Representative results of four experiments are shown by mean fluorescence intensity. (**B**) The mean fluorescence intensities of indicated molecules in each group were compared. Results are expressed as the mean ± SE (DMSO group: *n *= 4, TSA group: *n *= 4, *P-*value was *N.S*. in MHC class II, *P *= 0.035 in CD80, *P *= 0.023 in CD86, *P *= 0.012 in CD40). *N.S*., not significant.

Histone deacetylase inhibitors have been shown to block Th17 cells induction by altering dendritic cell function [27]. Thus, we hypothesized that TSA alters dendritic cell function and reduces Th17 cell generation in SKG mice and we examined the cell surface molecules on conventional dendritic cells in spleen. Spleen cells were used because the number of cells obtained from the lymph nodes was too small for FACS analysis. The mice in both the control and TSA-treated groups were killed on Day 35 (treatment for 21 days) and spleen cells were collected and analyzed using FACS, as described in the Materials and methods section. A gate was set on conventional dendritic cells, which are CD11c ^high ^and B220 ^negative ^cells, and the surface expression of various molecules was examined. There was no significant difference in the ratio of CD8α^+ ^and CD8α^- ^conventional dendritic cell subtypes (data not shown). In the CD8α^+ ^conventional dendritic cell subset, the expressions of CD86, CD80, and CD40 were significantly decreased in the TSA-treated group compared to the control group (Figure 4A, B), demonstrating the *in vivo *effects of TSA on conventional dendritic cells. In contrast, there were no significant differences in the expression of these molecules in the CD8α^- ^conventional dendritic cell subset (data were not shown). Thus, TSA predominantly affects CD8α^+ ^conventional dendritic cells *in vivo*.

### The effects of trichostatin A on cytokine production of zymosan A-treated dendritic cells *in vitro*

**Figure 5:**
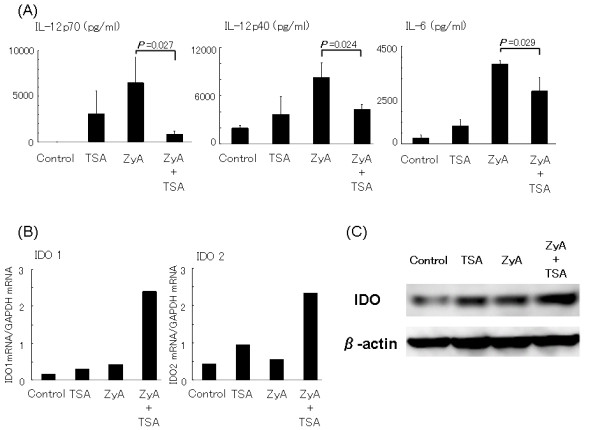
**The effects of trichostatin A on bone marrow-derived dendritic cells**. (**A**) The effects of trichostatin A on cytokine production by bone marrow-derived dendritic cells. Bone marrow-derived dendritic cells were generated from SKG mice and stimulated for 18 h with zymosan A and/or triclostatin A. The concentrations of IL-12p70, IL-12p40, and IL-6 in the supernatant were measured by an enzyme-linked immunosorbent assay. Values are presented as the mean ± SE (*n *= 3). Data are representative of two (IL-12p70) or three (IL-12p40 and IL-6) independent experiments with similar results (*P *= 0.027 in IL-12p70, *P *= 0.024 in IL-12p40, *P *= 0.029 in IL-6). (**B**) The effects of triclostatin A on indoleamine 2,3-dioxygenase mRNA expression by bone marrow-derived dendritic cells. Bone marrow-derived dendritic cells were generated from SKG mice and stimulated for 18 h with zymosan A (5 μg/ml) and/or trichostatin A (20 nM). IDO1 and IDO2 mRNA expression was measured by quantitative real-time polymerase chain reaction. Representative results of two independent experiments are shown. (**C**) The effect of trichostatin A on indoleamine 2,3-dioxygenase production by bone marrow-derived dendritic cells. Bone marrow-derived dendritic cells were stimulated with zymosan A (5 μg/ml) and/or trichostatin A (20 nM) for 48 h. Cell lysates were analyzed by Western blotting with anti- indoleamine 2,3-dioxygenase antibodies. The blot is representative of two independent experiments.

We found that TSA ameliorates severe arthritis in terms of both clinical and histological scores and modulates the conventional dendritic cell phenotype and Th17 cell generation *in vivo*. To further clarify the immune-regulatory functions of TSA, we examined the effects of TSA on the ZyA-treated dendritic cells *in vitro*. Bone marrow-derived dendritic cells were generated from SKG mice as described in Materials and methods. On Day 7, the cells were pulsed with ZyA (5 μg/ml), TSA (20 nM), or ZyA + TSA for 18 h. The cells and supernatants were collected. The IL-12p70, IL-12p40, and IL-6 cytokine levels expressed by ZyA-treated dendritic cells in the supernatant were significantly decreased in the presence of TSA, indicating that TSA inhibits the ZyA-induced production of these cytokines (Figure 5A).

### The effects of trichostatin A on IDO1 and IDO2 expression in bone marrow-derived dendritic cells

IDO1 and IDO2 expression in BM-DC were determined using real-time polymerase chain reaction. Both IDO1 and IDO2 are rate-controlling enzymes related to tryptophan metabolism and tryptophan depletion in the microenvironment has been reported to suppress cell proliferation [28,29]. Thus, IDO1 and IDO2 expressions in dendritic cells suppress the T cell reaction through tryptophan depletion. We examined the mRNA expression of IDO1 and IDO2 in BM-DC and found that TSA or ZyA alone induced IDO marginally, but the combination of TSA and ZyA markedly induced mRNA expression of IDO1 and IDO2 (Figure 5B). Western blot analysis confirmed the induction of IDO expression by BM-DC with the combination of ZyA and TSA at the protein level (Figure 5C). Protein expression levels of IDO were similar to the mRNA levels of IDO2.

### The effects of trichostatin A on cell surface molecules of bone marrow-derived dendritic cells

**Figure 6:**
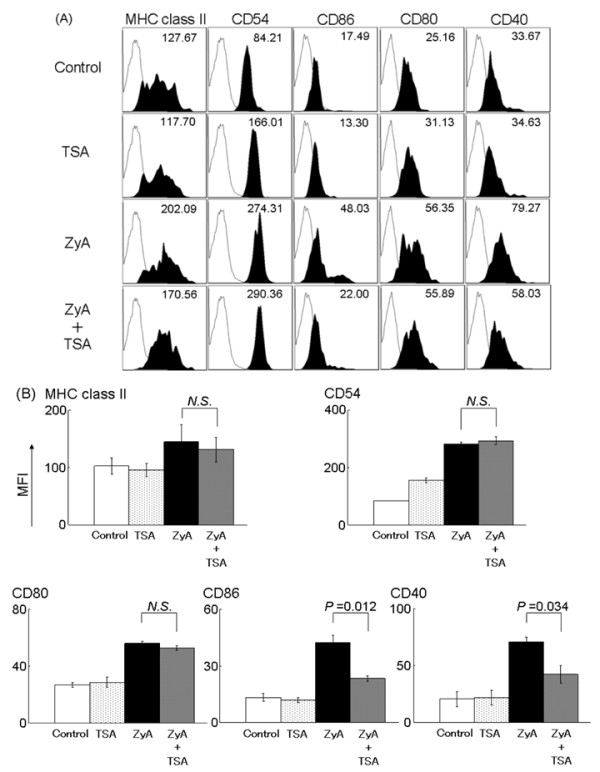
**The effects of trichostatin A on the phenotype of bone marrow-derived dendritic cells**. Bone marrow-derived dendritic cells were generated from SKG mice and incubated for 48 h with zymosan A and/or trichostatin A. Bone marrow-derived dendritic cells were stained for anti-MHC class II, anti-CD54, anti-CD86, anti-CD80, and anti-CD40. (**A**) The fluorescence activated cell sorting was shown by mean fluorescence intensity. Data are representative of three independent experiments. (**B**) The mean fluorescence intensity of each group was compared. Results are expressed as the mean ± SE of three independent experiments (*P-*values were *N.S*. in MHC class II, CD54 and CD80, *P *= 0.012 in CD86, *P *= 0.034 in CD40). *N.S*, not significant.

We analyzed cell surface expressions on BM-DC treated with ZyA (5 μg/ml), TSA (20 nM), or ZyA + TSA. After 48-h treatment, cell surface expressions of MHC class II, CD54, CD86, CD80, and CD40 on BM-DC were determined by FACS as described in Materials and methods. All these molecules were remarkably up-regulated after treatment with ZyA compared with those of the non-stimulated group (control). However, the expression of CD86 and CD40 were significantly down-regulated in the presence of TSA. Cell surface expressions of MHC class II, CD54, and CD80 did not differ between ZyA-treated and ZyA+TSA-treated BM-DC (Figure 6A, B). We failed to show the effect of TSA on CD80 expression *in vitro*, probably because the maturation stage may be different from dendritic cells *in vivo*.

### The effects of trichostatin A on dendritic cell-induced T cell proliferation

**Figure 7:**
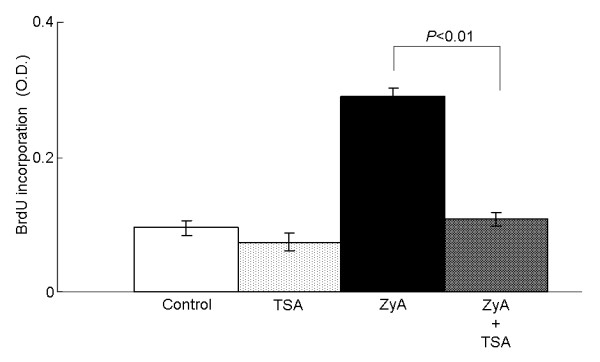
**The effects of trichostatin A on the T cell stimulatory capacity of dendritic cells**. Bone marrow-derived dendritic cells from SKG mice were treated with zymosan A and/or trichostatin A for 18 h, extensively washed, and used for the allo-mixed lymphocyte reaction to assess the T cell stimulatory capacity. Results are expressed as the mean ± SE of four independent experiments (*P *< 0.01).

We next tested the ability of TSA-treated dendritic cells to stimulate T cells by allo-mixed lymphocyte reaction. Bone marrow-derived dendritic cells from SKG mice were mixed with CD4^+ ^naïve T cells from C57BL/6 mice spleen. Pretreatment of BM-DC with ZyA alone augmented T cell proliferation, but co-treatment with ZyA and TSA resulted in reduced T cell proliferation compared to that with ZyA alone, indicating that TSA inhibited the ZyA-induced T cell stimulatory capacity of BM-DC (Figure 7). The results of the series of *in vitro *experiments indicated that TSA skewed dendritic cell function toward a tolerogenic phenotype.

### Therapeutic effects of trichostatin A on arthritis in SKG mice

**Figure 8:**
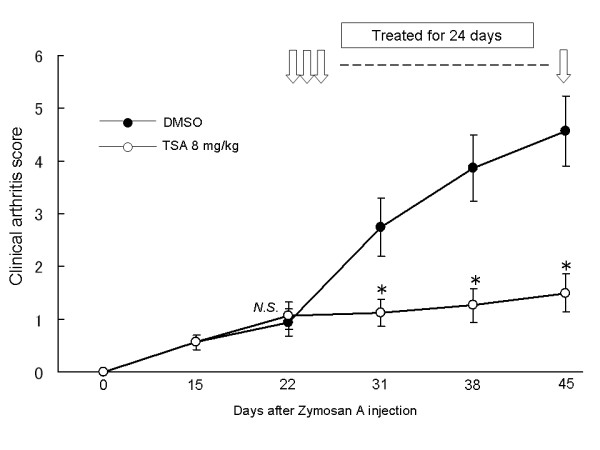
**Therapeutic effects of trichostatin A after the onset of arthritis in SKG mice**. SKG mice were treated with dimethyl sulfoxide or trichostatin A on Day 22, when the mean clinical arthritis score was nearly 1.0 for 24 days. Results are expressed as the mean ± SE (DMSO group: *n *= 8, TSA group: *n *= 8). * *P *< 0.05. *N.S*., not significant.

Finally, we examined the effect of TSA on SKG mice after the onset of arthritis. Arthritis was induced as described and TSA treatment was started on Day 22, when the arthritis scores had reached approximately 1. Trichostatin A treatment exhibited an inhibition of the worsening of clinical arthritis scores compared with DMSO, demonstrating the therapeutic effect of TSA on arthritis (Figure 8).

## Discussion

Our results have clearly shown that TSA ameliorates arthritis in SKG mice. The effects were characterized by a down-regulation of Th17 cells as well as up-regulation of Treg. We assumed that dendritic cells play a critical role in this model because it is well known that ZyA activates dendritic cells via the Dectin-1 and Toll-like receptor (TLR)-2 pathway [30,31]. Considering the significance of dendritic cells in determining Th cell differentiation, we examined the effects of TSA on dendritic cells *in vivo *and *in vitro *and concluded that TSA ameliorated arthritis, at least in part, by inhibiting dendritic cell activation.

Some reports have shown a therapeutic effect of HDAi on arthritis in mice; antibody-induced arthritis, collagen-induced arthritis, and adjuvant-induced arthritis have all been successfully treated with various HDAi [16-18]. Previous reports have shown that HDAi induce p21 in synovial fibroblasts, protect against cartilage apoptosis, inhibit matrix metalloproteinase production, and up-regulate Treg *in vivo *[17,32,33]. However, this is the first report to demonstrate that HDAi can ameliorate arthritis in a mouse model through regulating dendritic cells.

Our *in vitro *experiments indicated that HDAi skewed dendritic cell function to a tolerogenic-like phenotype. Zymosan A induced maturation of BM-DC, up-regulating expression of cell surface molecules, cytokine production, and T cell stimulation. When dendritic cells were stimulated with ZyA in the presence of TSA, a significant decrease was observed in the cytokine production, expressions of co-stimulatory molecules, and T cell stimulatory capacity, and a significant up-regulation of IDO gene and protein expression was also observed. Tolerogenic dendritic cells present antigens to antigen-specific T cells, but fail to deliver adequate co-stimulatory signals for effector T cell activation and proliferation[11]. Trichostatin A-treated dendritic cells *in vitro *are similar to tolerogenic dendritic cells in that they produce low levels of cytokines and high levels of IDO, but are different in that the expression of co-stimulatory molecules (CD80) is not markedly reduced. Thus, we consider that HDAi alter dendritic cells to a tolerogenic-like phenotype. Some previous reports have reported that histone deacetylase (HDAC) inhibition alters dendritic cell function when lipopolysaccharide was used to stimulate and differentiate dendritic cells [34]. We have found similar results using ZyA, which signals through Dectin-1 and TLR-2, instead of lipopolysaccharide, which utilizes TLR-4, illustrating that HDAC inhibition alters dendritic cell function regardless of the stimulation. Interestingly, activation of the Dectin-1 pathway has been shown to lead to the generation of Th17 cells, rather than Treg, through the syk-CARD9 pathway [35,36]. It is possible that TSA suppresses the Dectin-1 pathway in dendritic cells, resulting in decreased Th17 cell generation.

Dendritic cells regulate CD4^+^T cell differentiation and the immune response. Interleukin-12 is a key cytokine in Th1 cell differentiation and IL-6 is key in Th17 cell differentiation [37]. Tumor growth factor-β and retinoic acid induce Treg [38]. Our *in vivo *results demonstrated that TSA treatment markedly reduced Th17 cell population and slightly up-regulated Treg. Considering a larger effect of TSA on IL-17 production, TSA appears to have ameliorated arthritis in mice primarily by inhibiting the dendritic cell activation by ZyA because it has been shown that TSA and suberoylanilide hydroximic acid suppress Th17 cell differentiation by altering dendritic cell function [27]. Consistent with our results, some reports have shown the induction of Treg by HDAi treatment *in vivo *[33,39]. It is difficult to explain how TSA induces Treg *in vivo*. First, it is difficult to determine which subset of Treg, natural Treg or induced Treg, was derived by TSA in SKG mice [40-43]. Moreover, TSA might directly induce Treg through acetylation of Foxp3 or TSA might modulate dendritic cell function to indirectly induce Treg [44]. We have failed to determine the direct effects of TSA on Th cell differentiation *in vitro *because TSA suppressed naïve CD4^+ ^T cell proliferation so strongly as to prevent examination of the functional differentiation.

In mice, conventional dendritic cells that reside in lymphoid tissue can be separated into CD8α^+ ^and CD8α^- ^conventional dendritic cells [45,46]. We observed that TSA treatment *in vivo *significantly down-regulated co-stimulatory molecules on the CD8α^+ ^conventional dendritic cell subset, but not on the CD8α^- ^conventional dendritic cell subset. Furthermore, TSA tended to decrease the CD8α^+ ^conventional dendritic cell population compared to DMSO treatment, although the difference was not statistically significant (data were not shown). These results suggested that TSA mainly affected the CD8α^+ ^conventional dendritic cell population *in vivo*. CD8α^+^conventional dendritic cells are considered a more developed or activated form of CD8α^- ^conventional dendritic cells, because CD8α^+ ^conventional dendritic cells have been shown to more potently induce CD4^+^T cell proliferation and interferon-γ production compared with similarly activated CD8α^- ^conventional dendritic cells [47-49]. Recently, CD8α^+ ^conventional dendritic cells have been shown to produce IL-12p70 and induce antigen-specific Th17 and Th1 cells, resulting in the acceleration of collagen-induced arthritis [50]. Our results indicated that TSA treatment altered the CD8α^+ ^conventional dendritic cell phenotype to that of the tolerogenic CD8α^+ ^conventional dendritic cells and inhibited Th17 cell differentiation, leading to the suppression of arthritis in SKG mice, in which Th17 cells are critically involved [26]. Thus, we speculate that CD8α^+ ^conventional dendritic cells are one of the targets of the immunoregulatory effects of TSA.

It has been reported that vasointestinal peptide, IL-10, TGF-β, and vitamin D can induce tolerogenic dendritic cells. Histone deacetylase inhibitors are also useful for inducing tolerogenic dendritic cells in the treatment of rheumatoid arthritis, as we have shown in this report. SKG mice do not develop any arthritis in a specific pathogen-free environment, but develop severe arthritis after a single administration of ZyA, indicating that environmental factors contribute to the onset of arthritis [22]. Because microorganisms activate dendritic cells, targeting dendritic cell function is a rational way of regulating the autoimmune response triggered by microorganisms. It is noteworthy that HDAi-treated dendritic cells have been reported to be useful in the treatment of graft-versus-host disease in mice [51]. Thus, the prospects appear promising for dendritic cell-based cell therapy for rheumatoid arthritis using appropriate HDAi.

In conclusion, HDAC inhibition ameliorates arthritis in SKG mice, at least in part, by altering dendritic cell function into the tolerogenic phenotype. The HDAC protein family consists of at least 18 HDACs, including the sirtuin family of HDACs. Recently, HDAC9 was shown to be involved in Treg regulation [39], and HDAC11 was shown to be involved in immune tolerance by its effect on macrophage function [52]. Further understanding of HDAC functions in dendritic cells and the development of selective HDAi are expected to lead to novel therapies that target dendritic cells.

## Conclusions

Histone deacetylase inhibition changes dendritic cells to a tolerogenic phenotype and ameliorates arthritis in SKG mice.

## Abbreviations

2-ME: 2-mercapto-ethanol; APC: allophycocyanin; BM-DC: bone marrow-derived dendritic cells; DMSO: dimethyl sulfoxide; ELISA: enzyme-linked immunosorbent assay; FACS: fluorescence activated cell sorting; FITC: fluorescein isothiocyanate; HDAC: histone deacetylase; HDAi: histone deacetylase inhibitors; IDO: indoleamine 2,3-dioxygenase; PE: Phycoerythrin; PMA: phorbol myristate acetate; TLR: Toll-like receptor; Treg: regulatory T cells; TSA: trichostatin A; ZyA: zymosan A.

## Competing interests

The authors declare that they have no competing interests.

## Authors' contributions

KM participated in the conception and design of the data, carried out the acquisition of data, performed analysis and interpretation of data and drafted the manuscript. AM participated in the conception and design of the data, performed analysis and interpretation of data, and critically revised the manuscript. JS performed the analysis and interpretation of data. SK, MF, and FM carried out the acquisition of data. YM carried out the acquisition of data and performed the analysis and interpretation of data. SK participated in the conception and design, and revised the manuscript critically for intellectual content. All authors read and approved the final manuscript.
